# Limited syphilis testing for key populations in Zimbabwe: A silent public health threat

**DOI:** 10.4102/sajid.v37i1.385

**Published:** 2022-06-10

**Authors:** Mathias Dzobo, Tafadzwa Dzinamarira, Grant Murewanhema, Roda Madziva, Helena Herrera, Godfrey Musuka

**Affiliations:** 1 School of Health Systems and Public Health, Faculty of Health Sciences, University of Pretoria, Pretoria, South Africa; 2 Unit of Obstetrics and Gynecology, Faculty of Medicine and Health Sciences, University of Zimbabwe, Harare, Zimbabwe; 3 School of Sociology and Social Policy, University of Nottingham, Nottingham, United Kingdom; 4 School of Pharmacy and Biomedical Sciences, University of Portsmouth, Portsmouth, United Kingdom; 5 ICAP at Columbia University, Harare, Zimbabwe

**Keywords:** syphilis, HIV, key populations, testing, Zimbabwe

## Abstract

In this article, the authors discuss the problem of high prevalences of active syphilis amongst key populations (KPs) in Zimbabwe, in combination with low testing rates, partly because of a difficult legal and social environment for these populations. The article highlights the need to develop strategies to address the high prevalence of syphilis amongst KPs. The authors discuss requirements for addressing deficits in existing clinical services, predominantly primary care settings, in providing primary healthcare, including sexually transmitted infection (STI) management, to Zimbabwe’s KP communities and utility of point-of-care testing and self-testing and other innovations to improve testing uptake.

## Introduction

Syphilis, a sexually transmitted infection (STI), is caused by *Treponema pallidum* and is usually acquired by direct skin-to-skin contact with active primary or secondary lesions.^1,2^ It is an important cause of morbidity and mortality globally, despite marked advances in knowledge, prevention and treatment. Approximately 6 million new cases are reported annually globally in persons aged 15–49 years.^3^ Zimbabwe has one of the highest human immunodeficiency virus (HIV) prevalence in sub-Saharan Africa (SSA) at 12.8%, with 1.4 million people living with HIV (PLHIV) in 2019.^4^ Because of the overlap in risk behaviours, PLHIV are generally at an increased risk of acquiring STIs including syphilis. A 2015 national survey of the general population in Zimbabwe showed that active syphilis infection was higher amongst HIV-positive adults (2.9%) compared with their HIV-negative counterparts (0.4%).^4^ In the same national survey, amongst the general adult population, 2.7% were ever infected with syphilis, whilst 0.8% had an active syphilis infection.^5^

The risk factors for syphilis closely mirror those of HIV with a high risk of concurrent syphilis infections.^6^ Studies show that certain populations remain at substantial risk of HIV acquisition. These groups are termed key populations (KPs) and they include men who have sex with men (MSM), transgender women (TGW), people who inject drugs and sex workers. In the context of HIV infection, KPs are defined by the World Health Organization (WHO) as groups who because of specific risk profiles, are at increased risk of HIV regardless of the epidemic type or local context. Also, they often have legal and social issues related to their behaviour that increase their vulnerability.^7^

Compared with the general population, data on the prevalence of active syphilis amongst KPs are scarce in most SSA countries including Zimbabwe. A prohibitive environment, restrictive legislation, stigma and discrimination makes disclosure and/or self-identification as a member of a KP group by KPs very difficult, and hence accurate estimates of their numbers and disease patterns are not easily obtainable.^8^ A survey to determine the prevalence of active syphilis amongst MSM, TGW and genderqueer (GQ) individuals in Zimbabwe reported a high prevalence of active syphilis in Zimbabwe’s two major cities. The prevalence of active syphilis overall and amongst PLHIV was 5.5% and 10.1%, respectively, in Harare, and 5.6% and 11.0%, in Bulawayo.^9^ According to a 2019 report by the WHO, Zimbabwe has one of the highest prevalences of active syphilis amongst female sex workers (FSWs) with a prevalence of 30.3%.^10^ The prevalence of active syphilis amongst KPs in SSA is heterogeneous. A cohort study of 1978 FSWs in Rwanda reported a prevalence of 51.1% of active syphilis, with 27.4% of the cohort coinfected with syphilis and HIV.^11^ A cross-sectional study conducted in Togo in 2011 amongst 1106 FSWs reported that the seroprevalence of syphilis amongst FSWs was 2.2%. A cross-sectional survey of MSM in Togo reported a seroprevalence of 6.1% and a prevalence of 1.1% for active syphilis.^12^ In a cohort study of South African men and TGW who have sex with men, the prevalence of syphilis at baseline was 18.0% and 97.0% of the syphilis cases were clinically asymptomatic.^13^ The heterogeneity of active syphilis prevalence across SSA countries calls for country-specific programmatic interventions to address this public health problem of syphilis. It is also noteworthy that in all the studies where serology was used to classify syphilis cases as active, a substantial number of past treated syphilis cases may present with a low titre non-treponemal reactive result, which means that the prevalence data from the various studies may have overestimated the prevalence of ‘active’ syphilis infections.

Zimbabwe, like many other SSA countries, employs a syndromic approach to the management of genital ulcer diseases.^9,13^ Considering the high prevalence of asymptomatic disease, this approach is probably inadequate and may result in many missed opportunities to identify and treat infections. Firstly, in the context of syphilis management, it is important to note that syndromic management protocols do not manage secondary manifestations of syphilis, rather just STI syndromes. Secondly, the country lacks specific guidelines that target or cater for the health needs of KPs. Consequently, development partners and non-governmental organisations mostly promote and advocate for the health needs and rights of KPs.^14^

Syphilis may increase the risk of HIV transmission and acquisition by causing genital ulcers.^6,15^ The scale-up of syphilis testing amongst KPs in Zimbabwe is important to ensure the treatment of patients with active syphilis. In this perspective, we offer recommendations on how the government, key stakeholders in sexual and reproductive health can scale up testing of syphilis amongst KPs and link cases to care to avoid disease progression and onwards transmission. Granted, laboratories in low-resource settings often lack adequate infrastructure and trained personnel resulting in a limited diagnosis of STIs.^16^ A recent scoping review identified, self-testing, dried blood spots (DBS) and point-of-care (POC) testing as potential interventions to decentralise and scale-up syphilis testing amongst KPs.^17^ In this perspective, we discuss requirements for addressing deficits in existing clinical services, predominantly primary care settings, in providing STI healthcare to Zimbabwe’s KP communities and proffer our opinion on the utility of POC testing and self-testing and DBSs to improve testing uptake.

## Strategies to improve syphilis testing rates amongst key populations

### Point-of-care testing

Point-of-care testing involves conducting testing with results given within a short time, at or near the site of patient care by trained healthcare providers.^17^ Hard to reach KPs can be screened for syphilis and other STIs by deploying community health workers (CHWs) and nurses who use portable diagnostic devices to provide quick results. The timely release of these results can promote a screen and treat approach and prevent loss to follow up of screen positives. Different strategies to get KPs tested for STIs are well documented, for example, venue-based testing has been shown to increase the uptake of testing amongst KPs by offering testing at places they frequent, for example, bars and social clubs.^18^ China has leveraged on the rapid scale-up of HIV self-testing, to create an opportunity for integrated syphilis self-testing.^18^ In other places, peer-led POC testing models,^19^ financial incentives,^20^ venue based (e.g. in correctional facilities, drug rehabilitation centres) and mobile testing clinics^17^ have been used to engage subpopulation of KPs with varying feasibility and success.^19^ Commonly associated challenges with the use of POC tests is the issue of linkage to care. Additionally, quality assurance may be more difficult because of a greater number of and more dispersed sites.^21^ There have been concerns regarding the sensitivity of some of the devices,^22^ but when considering the potential number of KPs who can be reached through offering POC tests, the benefits can be thought to outweigh the concerns.

### Self-testing

The WHO has been advocating for the use of self-care interventions for sexual and reproductive health and rights (SRHRs). The WHO defines self-care as:

[*T*]he ability of individuals, families, and communities to promote health, prevent disease, maintain health, and to cope with illness and disability with or without the support of a healthcare provider.^23^

Self-care interventions can increase the participation of KPs in syphilis testing by offering privacy and facilitating end-user autonomy. A study conducted in Zimbabwe to assess the usability of syphilis self-testing in MSM reported high self-test usability and found that self-testing provided increased privacy, convenience and autonomy in comparison to hospital/clinic-based testing.^24^ In China, syphilis self-testing increased testing rates amongst MSM who had never tested for syphilis in the hospital (adjusted odds ratio [aOR]: 2.96; 95% confidence interval [CI]: 1.86–4.72).^18^ The similarities between HIV self-testing and syphilis testing may increase the acceptability of syphilis self-testing amongst KPs in Zimbabwe because some of the patients may be familiar with HIV self-test kits on the market, for example, the HIV OraQuick.^24^ It is important, however, to ensure the availability of facility-based confirmatory testing for patients who have reactive screening tests. To successfully implement this approach, there is a need to craft guidelines and education material specific for KPs on the correct use of devices and consider any concerns that might be barriers to the implementation of syphilis self-testing. A common limitation of self-testing is the lack of in-person counselling concerns about patients’ psychological welfare when receiving a positive result in isolation.^25,26^ To engage KPs in self-testing for syphilis, policymakers can use geosocial applications such as Facebook Places and Foursquare to recruit members of KPs who may want to have a self-test kit delivered to their homes or workplaces. When the recruitment is carried out by peer-led groups, this move has the potential to foster trust amongst KPs and increase their uptake in testing services. A key consideration of this strategy is to authenticate the public health message to distinguish them from spam, fraudulent and illegitimate messages.^27^

### Dried blood spot

Dried blood spot is a form of sampling where blood is blotted and dried on filter paper and sent to a laboratory for serological testing.^17^ The use of DBSs has gained traction over the years, playing an important role in early infant diagnosis (EID) of HIV and HIV viral load determination.^28^ The use of DBS is an important way of reaching hidden high-risk populations such as MSM, FSWs and TGW who have little use of health facilities by offering sample collection at venues of their convenience.^29^ Dried blood spot samples can be collected by trained non-medical professionals, for example, peers to KPs, this could increase trust and improve participation if the DBS samples are collected by people they know, it can also reduce the burden on medical staff in healthcare facilities.^30^ Once collected, DBS samples are easy to store and transport, a feature that allows the collection of samples in KPs living in remote areas that may not be prioritised under the traditional healthcare system. In addition, DBS samples allow for testing of multiple STI infections on the same card.^29^ In a study conducted amongst MSM in the Netherlands, more than 90% of the participants self-collected sufficient samples for HIV, hepatitis B virus (HBV) and syphilis testing on DBS.^29^ However, the use of DBS cards is not without limitations, compared with POC tests patients are not able to get results the same day they have been tested, this can create unnecessary anxiety amongst patients as they have longer waiting times for results. By comparison with available POC tests, one of the main limitations of using a DBS for diagnosis is the need for a post-test visit for counselling, which is associated with a risk of loss to follow-up.^31^ The use of diagnostic DBS samples has been ongoing in Zimbabwe for HIV viral load testing, the existing sample collection and storage, and sample referral systems can be leveraged to facilitate DBS use for testing syphilis amongst KPs in the country.

## Requirements to improve syphilis diagnosis and management in traditional healthcare settings

There is a need to address deficits in the existing clinical services, predominantly primary care settings, in providing primary healthcare, including STI management, to Zimbabwe’s KP communities. This is critical and will assist with linkage to care for the treatment of active syphilis cases. Firstly, health provider and/or health facility-based barriers could be addressed through training nursing and medical staff to improve their ability to manage KPs; such training would need to address stigma and common misconceptions. A study conducted in South Africa revealed that a sensitisation training increased healthcare workers’ knowledge and awareness about specific HIV-related health needs and psychosocial vulnerabilities of KPs, reduced moralising and judgemental attitudes and resulted in healthcare workers feeling more skilled to provide appropriate and sensitive services.^32^ Such training should emphasise the importance of opportunistic screening of KP members for syphilis/HIV when they attend routine clinical services. Appropriate training and education of clinical staff can go a long way to improve the quality of sexual health service provision. In addition to screening, the treatment for syphilis (injectable benzathine penicillin) is currently being given in either primary healthcare settings or hospitals. This means that KP members with active syphilis will need to engage with Zimbabwe’s health system and therefore underscores the need to make these public health services KP friendly and knowledgeable.

Secondly, there is a need to address the significant limitations of current point-of-care tests (POCTs) for syphilis, which are positive for life for those previously exposed to syphilis (i.e. the previously treated and the ‘active’ syphilis populations). To manage patients effectively, a non-treponemal test needs to be performed. For example, the non-treponemal tests and treponemal combined POCTs have been employed in antenatal care POC testing in low-income countries resulting in higher testing and treatment rates.^33^ This will be crucial to addressing the problem of significant overtreatment in KP, particularly in settings where the prevalence of syphilis is high if relying on treponemal POC tests alone.

Other considerations to scale-up testing amongst KPs involve task shifting, where the responsibility for certain clinical tasks is transferred, when appropriate, to less specialised healthcare staff, for instance, nurses and CHWs, instead of reserving the responsibility for doctors. These strategies decentralise sexual health services and improve the detection of more asymptomatic cases by introducing screening into primary care settings. Integration of syphilis and HIV testing at clinics is another strategy that can encourage testing of syphilis amongst KPs because HIV testing services are accessible to the majority of people in Zimbabwe. Digital technologies can also be leveraged to invite KPs for testing and to remind them when they miss appointments, social media can be an important tool to reduce stigma and discrimination, which can affect the health-seeking behaviours of KPs.

## Conclusion

Syphilis is an important public health problem and its high prevalence amongst the KPs in Zimbabwe warrants attention. Because of barriers in accessing healthcare services and intolerant social norms, limited number of KPs receive testing services. The use of POC platforms and self-testing can decentralise testing and meet the needs of KPs. Syphilis self-testing can be integrated with HIV self-testing to maximise benefits. There is a need to increase investment in innovative strategies that increase access to syphilis testing amongst KPs in Zimbabwe and ensure linkage to care. Partnering representative groups such as the Gays and Lesbians of Zimbabwe (GALZ) can go a long way in identifying sub-populations in different parts of the country who are in need of health services including syphilis testing.
